# Investigation of Frequency-Domain Dimension Reduction for A^2^M-Based Bridge Damage Detection Using Accelerations of Moving Vehicles

**DOI:** 10.3390/ma16051872

**Published:** 2023-02-24

**Authors:** Zhenkun Li, Yifu Lan, Weiwei Lin

**Affiliations:** Department of Civil Engineering, Aalto University, 02150 Espoo, Finland

**Keywords:** bridge health monitoring, damage detection, indirect method, dimension reduction

## Abstract

Recent decades have witnessed a rise in interest in bridge health monitoring utilizing the vibrations of passing vehicles. However, existing studies commonly rely on constant speeds or tuning vehicular parameters, making their methods challenging to be used in practical engineering applications. Additionally, recent studies on the data-driven approach usually need labeled data for damage scenarios. Still, getting these labels in engineering is difficult or even impractical because the bridge is typically in a healthy state. This paper proposes a novel, damaged-label-free, machine-learning-based, indirect bridge-health monitoring method named the assumption accuracy method (A${}^{2}$M). Initially, the raw frequency responses of the vehicle are employed to train a classifier, and K-folder cross-validation accuracy scores are then used to calculate a threshold to specify the bridge’s health state. Compared to merely focusing on low-band frequency responses (0–50 Hz), utilizing full-band vehicle responses can significantly improve the accuracy, meaning that the bridge’s dynamic information exists in the higher frequency ranges and can contribute to detecting bridge damage. However, raw frequency responses are generally in a high-dimensional space, and the number of features is much greater than that of samples. To represent the frequency responses via latent representations in a low-dimension space, appropriate dimension-reduction techniques are therefore, needed. It was found that principal component analysis (PCA) and Mel-frequency cepstral coefficients (MFCCs) are suitable for the aforementioned issue, and MFCCs are more damage-sensitive. When the bridge is in a healthy condition, the accuracy values obtained using MFCCs are primarily dispersed around 0.5, but following the occurrence of damage, they increased significantly to 0.89–1.0 in this study.

## 1. Introduction

Since the beginning of this century, bridge-health monitoring has been a concern due to the rapid deterioration of civil infrastructures in several countries. It was reported that in Europe, the majority of transportation bridges constructed after 1945 were planned for a 50–100-year service life. The design laws in the previous century might not be suitable for the increasing traffic loads nowadays [1]. As a result, monitoring a bridge’s health condition and providing practical maintenance measurements are of great importance for engineers [2,3,4,5]. As a key task in bridge-health monitoring, damage detection techniques interest many researchers worldwide. The traditional method of inspecting bridge structures entails visual monitoring by skilled engineers [6,7], which is often time-consuming and even dangerous as bridge spans and heights increase. In addition, some common forms of damage, such as internal cracks and corrosion, cannot be seen directly and can challenge the monitoring results. Vibration-based approaches can offer fresh ideas to identify the bridge’s damage in order to overcome these drawbacks [8,9,10]. Typically, for the vibration-based methods, sensors are mounted to the bridge directly to collect the stream accelerations (say, the direct method). The health of the bridge is then evaluated by examining its vibration modes, which include natural frequencies, modal shapes, and damping ratios. However, the direct method typically necessitates the installation of numerous sensors on the bridge to create a sensing system, which is costly [11]. Additionally, installing sensors may cause traffic pauses once the bridge is constructed, which could result in losses in revenue [12].

In 2004, Yang et al. [13] proposed to monitor the bridge’s modes using the passing vehicle’s vibrations, which was named the indirect method (also vehicle scanning method or drive-by method). The indirect method is more cost-effective for monitoring the bridge because it only requires a few sensors to be attached to moving vehicles. In addition, it was discovered that the ongoing traffic helped to distinguish modes of the bridge [14]. Therefore, when the indirect method is employed, no traffic pauses are required. Since the sensors are installed on the vehicle, it becomes easy to maintain the sensors once faults are found. In the reference [13], the authors successfully identified the bridge’s fundamental frequency using a mass-spring model, and later the idea was verified by Lin and Yang in a field test [15] using a tractor-trailer system on Da-Wu-Lun Bridge in Taiwan. Thereafter, identifying the bridge’s natural frequencies from the vehicle’s accelerations was also investigated using numerical simulations [16,17,18,19], laboratory experiments [20,21,22], and field tests [23,24,25,26,27]. Apart from the bridge’s frequencies, it was found that modal shapes [28,29,30,31,32,33] and damping ratios [34,35,36,37,38] could also be extracted from the passing vehicle’s vibrations. However, in current studies, the indirect method normally depends on same driving traces, constant speed, and so on, which were challenging in practical engineering. In addition, it was reported that the bridge’s modes were not quite sensitive to damage [39]. New damage indicators are therefore required to identify the bridge’s damage.

Due to the developments in computer science, machine learning (ML) techniques have flourished in recent decades [40]. Researchers are attracted by their good properties, such as requiring no field knowledge and having good learning capabilities. They have been increasingly employed in bridge-health monitoring [41,42,43,44]. In recent years, researchers noticed that the sound characteristics of ML techniques were suitable for the indirect method when the vehicle’s vibrations were utilized. Cerda et al. (2014) [45] proposed to classify healthy and different damage scenarios of the bridge using support vector machine (SVM) models. The bridge’s damage was simulated by changes in the support conditions, an increase in the damping, or a localized mass increase. Results showed that when the vehicle’s vibrations were utilized, classification accuracy could be as good as or even better than the accuracy when the bridge’s accelerations were directly used. Malekjafarian et al. (2019) [46] employed artificial neural networks (ANNs) to identify the bridge’s damage. Two ways were proposed using the vehicle’s time-domain and frequency-domain responses, respectively. It was reported that the occurrence of damage could be identified, and the reference to damage severity was also provided. Furthermore, to improve the proposed method, Corbally and Malekjafarian (2021) [47] utilized the contact-point (CP) frequency responses to feed ANNs. It was found that CP responses were superior to accelerations when identifying the bridge’s damage. The proposed method was tested robustly with different vehicle speeds, ambient temperatures, and road roughness levels. Locke et al. (2020) [48] presented a study to predict bridge damage severity directly from the frequency responses of a passing vehicle using convolutional neural networks (CNNs). The results indicated that the proposed model could automatically discard some complicating factors, such as different climates, temperatures, and damage patterns, and was able to predict the bridge’s damage correctly. Feng et al. (2021) [49] proposed a damage detection method based on the k-nearest neighbors (KNN) algorithm that employs instantaneous forced frequencies to localize and quantify the bridge’s damage. It was found that the damage’s degree and position could be identified in optimal cases, but the precision was relatively low near supports. Hajializadeh (2022) [50,51] proposed a transfer learning-based drive-by bridge monitoring framework. The weights were adopted from a pre-trained CNN entitled GoogLeNet, and the raw train-borne accelerations were used as damage-sensitive features. Remarkable accuracy was obtained when 30% of the dataset (including healthy and damaged cases) was utilized for testing. However, in current studies, the damage detection methods generally require labels indicating damage to the bridge. In practical engineering, the bridge will mostly be in a healthy state; obtaining labeled vehicle vibration data for damaged bridges is often difficult or cannot be achieved. To respond to this challenge, some researchers proposed using unsupervised methods to extract damage-sensitive features from the vehicle’s vibrations. For instance, using the k-means, Yang et al. [52] automated the bridge frequency identification process in the frequency domain. Sarwar and Cantero [53] adopted the deep autoencoder (DAE) to extract the mean absolute error (MAE) values for damage indicators from the vehicle’s time-domain accelerations. Results showed that the damage could be identified, and its severity could be reorganized from the distribution of reconstruction errors. Even though the unsupervised method can get good results, it usually requires a large number of “healthy” cases to learn their features, which may challenge their applications in engineering.

A machine learning-based indirect bridge-health-monitoring method named the assumption accuracy method (A${}^{2}$M), is proposed in this paper. It does not require data when the bridge is damaged and thus is more practical in engineering applications. Apart from detecting damage, this paper also discusses accuracy values when the bridge is healthy, and the threshold is provided for determining the bridge’s healthy condition. To improve the detection efficiency and precision, the vehicle’s raw frequency responses are mapped into low spaces, and different dimension reduction techniques are explored. The robustness of the proposed method was tested by various damage scenarios with various types of vehicles and bridges. The remainder of this paper is organized as follows: Section 2 introduces the theoretical foundation of the proposed method. The laboratory setups and artificial damage cases are provided in Section 3. Section 4 explores damage-detection results using raw frequency responses and the capabilities of different dimension reduction techniques. Finally, conclusions and future work of this paper are provided in Section 5.

## 2. Theories for Damage Detection

**Figure 1:**
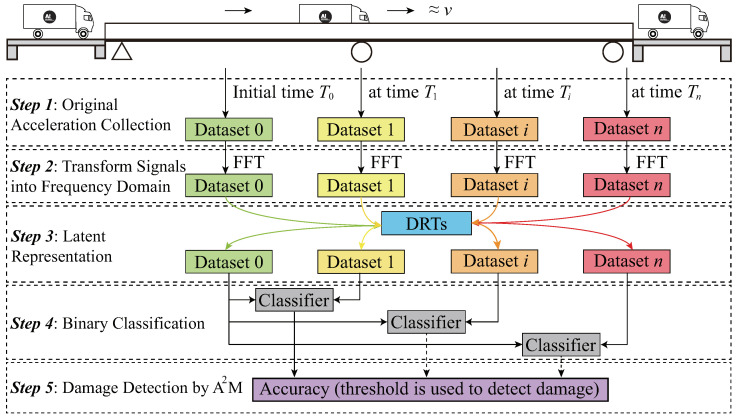
Schematic workflow of the proposed method.

In current studies of the indirect method, typically only low-frequency responses (0–50 Hz) are investigated—for example, extracting the bridge’s natural frequencies from the vehicle’s vibrations. However, on the one hand, engineering applications may fail since the identification of the bridge’s dynamic properties rely on the vehicle models [16,54,55]. On the other hand, in the vehicle’s frequency-domain responses, not only the low-frequency responses (0–50 Hz) but the frequency responses in the higher range (>50 Hz) contain the bridge’s damage-sensitive information [56], which is generally ignored by researchers. This paper investigates the full-band frequency responses and analyzes different dimension-reduction techniques (DRTs) to quickly determine the bridge’s health state by employing the vehicle’s vibrations. The classifier we selected is the logistic regression (LR) algorithm, and five DRTs were explored. They were principal component analysis (PCA), uniform manifold approximation and projection (UMAP), multidimensional scaling (MDS), stacked autoencoder (SAE), and mel frequency cepstral coefficients (MFCCs). Figure 1 shows the schematic workflow of the proposed damage detection framework. There are five steps: (1) original acceleration collection, (2) transform signals into the frequency domain, (3) latent representation, (4) binary classification, and (5) damage detection by A${}^{2}$M. All steps are introduced in detail below.

### 2.1. Acceleration Collection

In this study, accelerometers were attached to the vehicle’s front and rear axles, and its vertical accelerations were collected when passing the beam. Only the accelerations measured after the entire vehicle was on the bridge were used for analysis, in order to minimize irrelevant signals. Suppose that the front and rear wheels of the vehicle enter the bridge at $t_{f0}$ and $t_{r0}$, and they leave the bridge at $t_{f1}$ and $t_{r1}$. Then, the signals between $t_{r0}$ and $t_{f1}$ are employed, which can be represented by Equation (1):

(1) $$\Delta t^{p}=t_{f1}-t_{r0}$$

where $\Delta t^{p}$ means the vehicle’s passing time when its double axles are both on the bridge. The bridge is assumed to be in good condition at initial time $T_{0}$ (early stages after construction). The vehicle is expected to make multiple trips across the bridge (named $N_{0}$ runs). In order to include as many influence factors as possible during this time, the vehicle may run at slightly varying speeds and road traces on the bridge. These data will be labeled as “healthy”. At the time $T_{i}$ (may be months or years after $T_{0}$), the same vehicle needs to pass the bridge again, $N_{i}$ times. $N_{i}$ is expected to be equal to $N_{0}$ and greater than 50 for training the classifier. However, if $N_{i}<N_{0}$, $N_{i}$ runs need to be randomly selected from the $N_{0}$ “healthy” runs for the later binary classification. Alternatively, the random selection will be carried out in $N_{i}$ runs if $N_{i}>N_{0}$. The primary goals of the aforesaid procedure are to (1) avoid data imbalance while performing binary classification and (2) lessen the impacts of non-damage factors on the detection results.

### 2.2. Signal Transformation

After the accelerations at different times $T_{0}$ and $T_{i}$ are collected, they will be transformed to frequency signals using fast Fourier transformation (FFT) to obtain the vehicle’s frequency responses. The Hann window is used during this process to avoid the spectrum leakage phenomenon. The lengths of different serials of signals differ, since the vehicle’s passing speeds vary regardless of time $T_{0}$ or $T_{i}$. As a result, every run will have a different frequency resolution in the frequency domain. To adjust this, time-domain signals are followed by padding zeros to ensure that all data serials are the same length. Then, the lengths of the padded signals represented in the frequency domain will be equal. The padding zero techniques will increase FFT resolution in the frequency domain without affecting the dynamic information of the original signals. Following the collection of the vehicle’s frequency responses, a classifier can be fed with the responses at each frequency point to determine the health state of the bridge.

### 2.3. Dimension Reduction

Raw frequency responses from Section 2.2 are acceptable for damage detection directly. However, huge amounts of features sharply lower detection efficiency. Additionally, ambient noises can play a negative role in damage detection. To address this, the capabilities of five DRTs in extracting key information from original responses are explored, as introduced below.

One of the most popular DRTs is the PCA [57], which maps *n*-dimension data into *k* principal components while maintaining important information. Correlated variables can be eliminated by PCA, making representations clear and independent for decision making.

Another two nonlinear dimension reduction methods are UMAP and MDS [58,59]. UMAP has better preservation of the global structure, more understandable parameters, and superior run-time performance than PCA. For MDS, it uses geometric coordinates to determine the distances between each pair of points. It is a suitable method for preserving high-dimensional data’s global and local structures.

SAE stems from unsupervised deep learning [60], which compresses the input into latent representations in the autoencoder’s bottleneck. It is not constrained to optimize a convex objective function and has many degrees of freedom [56].

**Figure 2:**
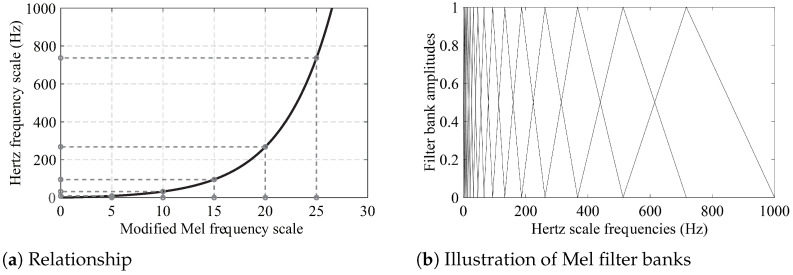
Relationship between modified Mel- and Hertz-scale frequencies and illustration of Mel filter banks.

The idea behind MFCCs came from acoustic recognition and was first intended to mimic how the human ear works [61]. The key advantage of MFCCs is the concentration on the low-frequency ranges when it comes to SHM rather than the high-frequency band, where signals may be poorly noised. The modified relationship between Hertz and Mel frequency scale is indicated in Equation (2) [62]:

(2) $$f_{Hertz}=5\left(e^{f_{Mel}/5}-1\right)$$

where $f_{Hertz}$ is the Hertz-scale frequency and $f_{Mel}$ is the Mel-scale frequency. Figure 2a depicts the modified relationship between Hertz- and Mel-scale frequencies. The dimensionality of the raw frequency responses can be decreased by using different numbers of Mel filter banks. Figure 2b shows a visualization of 15 Mel filter banks in the 0–1 kHz range.

### 2.4. Binary Classification

**Figure 3:**
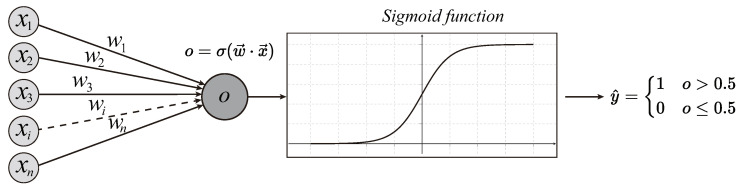
Illustration of logistic regression.

After the original accelerations are preprocessed and denoted by latent representation using different DRTs, dataset 0 and dataset *i* at different times (shown in Figure 1) are expected to be fed into a classifier. Then, the classifier will learn the two datasets’ features and try its best to determine the bridge’s healthy state. In this paper, the LR is employed for binary classification. Figure 3 plots the basic concept of LR.

In Figure 3, the features $x=\{x_{1},x_{2},\ldots ,x_{n}\}$ denote the latent representation of the vehicle’s frequency responses. $w=\{w_{1},w_{2},\ldots ,w_{n}\}$ are the weights of all features. σ is the activation function, and it is a sigmoid function for binary classification problems. After activation, the output $\hat{y}$ can be obtained. If $\hat{y}>0.5$, the datapoint (one run at time $T_{0}$ or $T_{i}$) will be determined as class 1, and otherwise class 0. Classes are pre-allocated to datasets. At the initial time, $T_{0}$, $N_{0}$ runs are labeled as class 0, and $N_{i}$ runs at a later time $T_{i}$ will be labeled as class 1.

### 2.5. Damage Detection

In the above four steps, if the bridge is damaged, the accuracy of binary classification is expected to be relatively high. However, in practice engineering, the dataset clearly stating that the bridge is damaged cannot be obtained. At time $T_{i}$, when the dataset *i* is obtained, we cannot know the bridge’s condition, so it cannot be labeled as damaged.

To solve the above problem, the A${}^{2}$M method is proposed in this paper. When a new dataset, e.g., dataset *i*, is obtained, all $N_{i}$ runs will be assumed as damaged (the bridge may not be damaged indeed), namely, class 1 for binary classification. Then, the binary classification accuracy will be checked, and as references for determination of the occurrence of damage in the bridge. Once the bridge is damaged, the classification accuracy is expected to be high (close to 1.0) because the bridge’s dynamic information related to the damage is contained in the vehicle’s vibrations. However, if the bridge remains in good condition, the accuracy will become low (near 0.5), since no bridge damage information can be detected from the vehicle’s vibrations. When applying the proposed A${}^{2}$M method, the following points need to be noted:

1. At the initial time when the bridge is in a healthy state, the vehicle is expected to run over the bridge numerous times to collect enough data, representing that the bridge is in its healthy condition. During this period, the influence factors need to be included as much as possible, such as different environmental noises, driving traces, and vehicle speeds.
2. When doing binary classification, the samples of classes 0 and 1 need to be nearly equal to avoid data imbalance problems, as discussed in Section 2.1.
3. To calculate the classification accuracy, k-folder cross-validation (CV) is expected to be used, rather than just one-time validation. As in the CV process, the whole dataset (including dataset 0 and *i*) is randomly divided into k sections. This k-folder CV process needs to be executed many times to avoid possible occasional high or low accuracy.
4. The classifier cannot be an extremely strong one because these strong classifiers can identify the minor differences between two datasets, even if the bridge is in its “healthy” state all the time. The differences these classifiers find may be irrelevant to damage, and simply noisy features. It is shown that in high-dimensional space, the vehicle’s frequency responses are linearly separable [45]; thus, some linear classifiers, such as the support vector machine (SVM) with a linear kernel, and weak classifiers such as naive Bayes (NB), k-nearest neighborhood (KNN), and decision tree (DT) without boosting techniques are recommended.

## 3. Experiments

In this section, the experimental vehicle bridge interaction (VBI) setups are introduced. To verify the proposed idea, the vehicle was simulated by a scaled truck with different weights and two kinds of beams, simply supported beam (SSB) and continuously supported beam (CSB), were employed. The following provides a detailed description of the vehicle and bridge models.

### 3.1. Vehicle Bridge Interaction Models

#### 3.1.1. Vehicle Models

**Figure 4:**
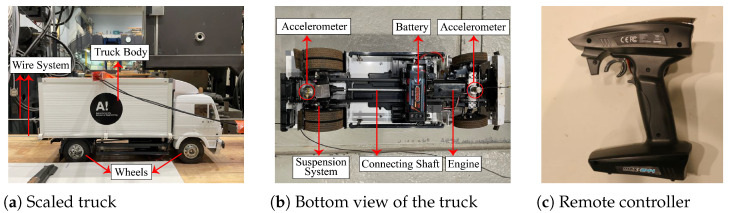
Vehicle model.

The vehicle used in the experiment was a carefully scaled truck model, as shown in Figure 4a. The type of truck model was the Tamiya Mercedes-Benz 1850L. Tamiya is a Japanese manufacturer that is famous due to its accurate scaled details of all vehicle components. The scale ratio of this truck is 1:14. The normal truck’s mass is 4.305 kg (named v0). To simulate a heavy vehicle, an additional 5.157 kg mass was added to the truck’s trunk (named v5). The guide-wire system was employed to keep the truck running on a relatively straight trace and to not hit the flange of the beam. However, the wire was not extremely tight because a tight wire would limit the vibrations of the vehicle. Due to the loose wire used, the truck’s passing traces may not be exactly the same when it passes the beam many times.

The scaled truck’s bottom view is shown in Figure 4b. We can see that the truck has a scaled suspension system, engine, connecting shaft, etc. It can be driven by a 540-brushed electric motor powered by a Tamiya Ni-MH 7.2 V–3000 mAh battery. The engine’s noises can have a great influence on acceleration collection, which is similarly unavoidable in practical engineering. Two accelerometers, made by Brüel & Kjær (type 4371), are attached to the vehicle’s front and rear axles, as shown in Figure 4b. The vehicle can be controlled by the remote controller shown in Figure 4c. Due to the battery’s capability, the vehicle’s speed varies slightly for different runs.

#### 3.1.2. Bridge Models

**Figure 5:**
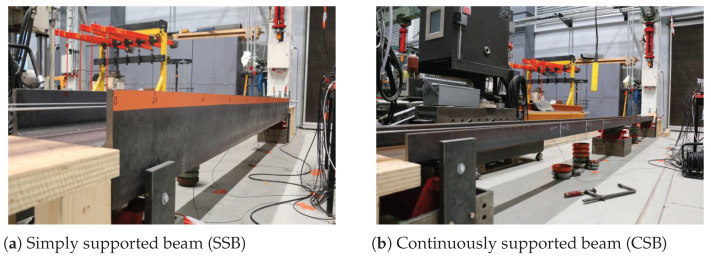
Bridge models.

**Figure 6:**
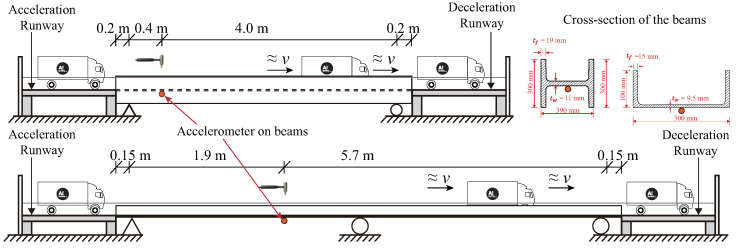
Illustration of beams.

**Figure 7:**
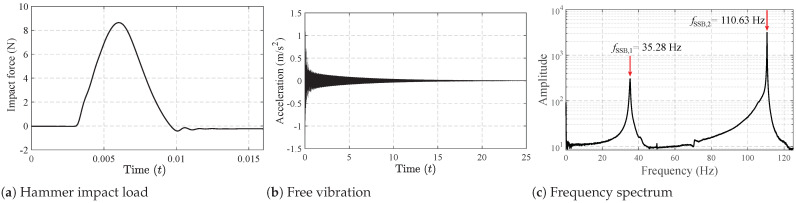
Free-vibration tests of the SSB model.

**Figure 8:**
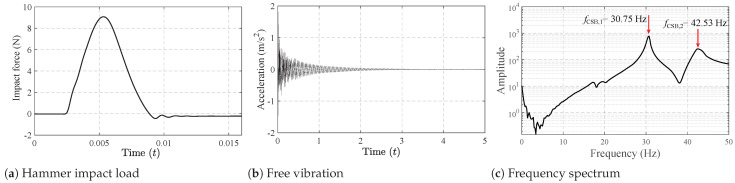
Free-vibration tests of the CSB model.

For the bridge, two models, SSB and CSB, were employed in the experiment. The basic parameters of the two beams are listed in Table 1. Two beams in the laboratory are shown in Figure 5, and their layouts can be found in Figure 6. To drive the truck at a relatively constant speed when passing the beams, acceleration and deceleration runways were utilized at their ends. Positions of the accelerometer on the SSB and CSB models can be found in Figure 6, and they were attached to the bottoms of the beams. An impact hammer was employed to impact the beam exactly on the other side of the beam’s web. The impact forces are shown in Figure 7a and Figure 8a for two beam models, and the beams’ acceleration responses can be found in Figure 7b and Figure 8b. By utilizing the FFT, we can get the natural frequencies of the two beam models, as shown in Figure 7c and Figure 8c. We can see that the SSB model’s first two frequencies were 35.28 and 110.63 Hz, and the same parameters of the CSB model were 30.75 and 42.53 Hz. Using the above vehicle and bridge models, we could obtain the following four scenarios:

(1) Scenario 1: SSB model + normal vehicle (v0).
(2) Scenario 2: SSB model + heavy vehicle (v5).
(3) Scenario 3: CSB model + normal vehicle (v0).
(4) Scenario 4: CSB model + heavy vehicle (v5).

**Table 1:** Bridge model parameters.

Model	Type	Cross-Section Area/$m^{2}$	Support Length/m	Span Length/m	Mass/kg
SSB	HEA 400	$1.59\times 10^{-2}$	0.20	4.0	550.00
CSB	UPE 300	$5.66\times 10^{-3}$	0.15	5.7	248.64

**Figure 9:**
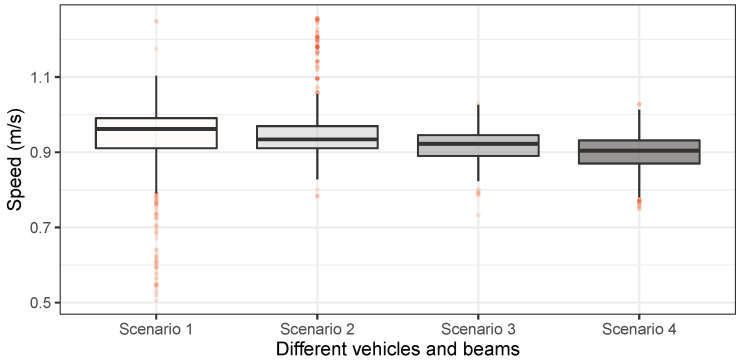
Speed distribution.

The vehicle’s mass is usually less than 5% of the bridge [12,63]. In this experiment, the ratios for the SSB model were 0.78% (v0) and 1.72% (v5), and for the CSB model, they were 1.73% (v0) and 3.81% (v5). Those ratios are reasonable for simulating a practical VBI system. The vehicle’s speed is plotted in Figure 9 for different scenarios. It can be seen that the scaled truck’s speed was between 0.85 and 1.1 m/s. When the extra mass was added to the vehicle, its speed’s mean value became slightly slow compared to the normal truck. Since the vehicle’s high-frequency responses were also investigated in this study, the sampling frequency for the vehicle and beams was set to 10 kHz, which is much higher than normally used ones. The experiment was accomplished in the structural laboratory at Aalto University with normal environmental noises.

### 3.2. Damage Cases

**Figure 10:**
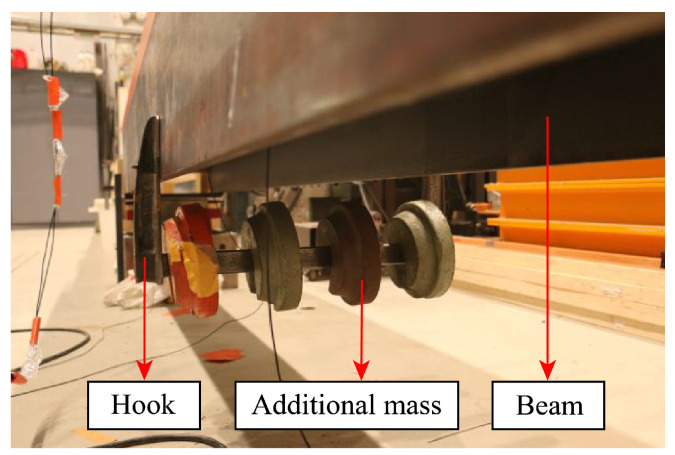
Adding a 20 kg mass to the SSB model.

Bridge damage can typically be simulated by local stiffness loss, which will induce a decrease in the structural natural frequencies. By achieving similar results for the bridge’s frequency responses, a practical way to simulate the bridge’s damage is to attach additional mass to it [45,56,64,65]. Since the bridge’s natural frequencies are negatively related to its mass matrix, once the mass increases, its frequencies will decrease, causing similar effects to the occurrence of damage. As this study was meant to investigate the bridge’s dynamic information included in the vehicle’s vibrations, and frequency responses were utilized as damage-sensitive features, the additional mass method was able to be utilized for simulating the bridge’s damage. An illustration of adding a 20 kg mass to the simply supported beam is shown in Figure 10.

Table 2 shows all cases in the experiment when the bridge was healthy or had artificial damages. We simulated several different damage cases for both SSB and CSB models. After the different masses were added to the bridge models, the bridge’s modal parameters were changed. The bridge’s fundamental frequency could be identified using the accelerometer attached to the bridge (direct method), as shown in Figure 6. The bridge models’ fundamental frequencies in different cases are listed in Table 3. It can be seen that compared to the intact bridges, the fundamental frequency of a bridge with added mass decreasd by 0.74% to 15.64%.

As the objective of this study was to detect the occurrence of damage using a classifier, all damage cases with different damage positions or degrees can be uniformly regarded as “damaged”, and they are named SC3 (or CC3), where the first “S” means SSB (the first “C” in CC3 means CSB). However, in practice, we do not know whether the bridge is damaged or not in advance. To suit the A${}^{2}$M and avoid the data imbalance problem, half of the “healthy” runs were assumed as “damaged”, and they were named SC2 (or CC2). Here is an example of scenario 1. There were 568 runs when the bridge was healthy, and half of them (284 runs) were randomly selected as SC1, and the other half (284 runs) were assumed as damaged and named SC2. Then, the classifier was utilized to do a binary classification and get the accuracy using raw frequency responses or latent representations. For SC3, there were 66 + 79 + 66 = 211 runs. Then, 211 runs were randomly selected from all 568 “healthy” runs as SC1. Thus, we can see that runs in SC1 can be different for different problems. The number depends on how many runs we will measure in a future time, namely, at time $T_{i}$, as introduced in Section 2.1. To summarize, for different scenarios, the binary classification problems are:

- Scenario 1: SC1 vs. SC2 (the bridge remains healthy), and SC1 vs. SC3 (the bridge is damaged).
- Scenario 2: SC1 vs. SC2 (the bridge remains healthy), and SC1 vs. SC3 (the bridge is damaged).
- Scenario 3: CC1 vs. CC2 (the bridge remains healthy), and CC1 vs. CC3 (the bridge is damaged).
- Scenario 4: CC1 vs. CC2 (the bridge remains healthy), and CC1 vs. CC3 (the bridge is damaged).

## 4. Results and Discussion

In this section, the experimental results are discussed in regard to the introduced experimental devices, vehicle, and bridge models. Firstly, the vehicle’s frequency responses are visualized in a low-frequency range (0–50 Hz). Then, the raw full-band frequency responses are explored using the LR classifier. The LR model in the scikit-learn package [66] was used with a penalty term of $l_{2}:C=1.0$, where *C* is a hyper-parameter controlling how strong the penalty term is. Smaller values specify stronger regularization. In this study, the authors tested the hyper-parameter *C*, and its selection has a small effect on the classification accuracy.

### 4.1. Damage Detection Using Frequency Responses

**Figure 11:**
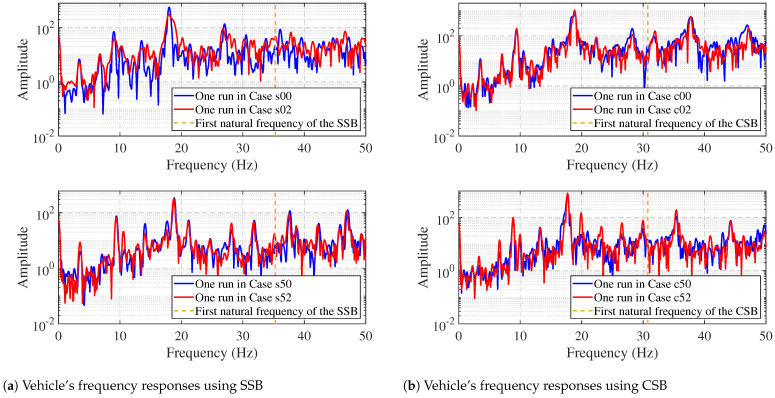
Vehicle’s frequency responses at 0–50 Hz.

Figure 11 has the vehicle’s frequency responses in 0–50 Hz. It can be seen that for different beams and vehicles, the bridge’s fundamental frequency is polluted in the vehicle’s frequency responses when the bridge is healthy (blue lines). Additionally, we can see that even when the bridge’s damaged (red lines), we cannot determine the bridge’s condition visually, as no peaks show the bridge’s fundamental frequency. Therefore, damage detection with prior knowledge in a low-frequency range is not achievable.

**Figure 12:**
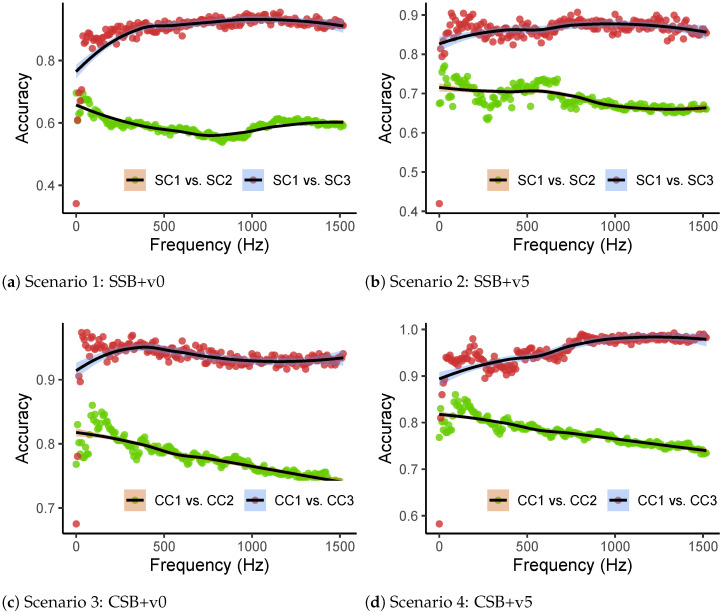
Classification accuracy with different frequency ranges.

Due to the above issues, a machine learning classifier was employed to detect the bridge’s damage using raw frequency responses. The advantage of machine learning is that it can detect damage-sensitive features automatically. It can detect minor changes in the vehicle’s responses before and after the bridge is damaged [64]. This section shows the binary classification results when the raw frequency responses are used, which means step 3 is not executed in Figure 1. Different vehicles and beams were utilized, and the accuracies of LR models with increasing features (frequency responses) are plotted in Figure 12 (frequencies greater than 0–1500 Hz are not plotted because more features will not improve classification accuracy). In Figure 12, green and red dots mean accuracy values in relation to the frequency-response range. The black lines are smoothed curves using the LOESS (locally estimated scatterplot smoothing) method, and the color bands around them are 95% confidence intervals.

It can be seen in Figure 12 that for the SSB model, when the selected frequency range increases, the classification accuracy of SC1 vs. SC3 increases (>0.8), but the results of SC1 vs. SC2 always show relatively low accuracy—e.g., around 0.6 for scenario 1 and 0.7 for scenario 2. We can see that when the bridge is healthy, the accuracy is not always near 0.5. This is because the vehicle’s signals are strongly influenced by multiple factors, such as the engine, environmental noises, and passing traces. When the classifier attempts to classify two classes (healthy and damaged bridges), it may detect other influence factors present in the frequency domain, resulting in higher accuracy. Despite the classification being influenced by other factors, it is clear that the bridge’s deterioration is a crucial factor that can result in more accuracy than when the bridge is healthy. Distinct divergence can be observed in Figure 12 for when the bridge is healthy or damaged. Similarly, for the CSB model, we can see that when the bridge is damaged (CC1 vs. CC3), the accuracy can be higher than 0.9. When the bridge is still healthy (CC1 vs. CC2), the accuracy will be lower than 0.9. Additionally, we can see that when a heavy vehicle (v5) was utilized, the accuracy of the classification was higher than when the normal vehicle (v0) was used. Thus, a heavier car is recommended for damage detection of indirect bridge-health monitoring in practical engineering.

To determine the threshold for damage detection for general cases in engineering, the 5% and 95% quantiles calculated for all scenarios are shown in Table 4. It can be seen that even when the bridge is in its healthy state, the accuracy can reach 0.74. This is because when the raw frequency responses are utilized, the accuracy is prone to be affected by influence factors. For example, when two groups in the “healthy” runs are selected, it is easy to group them with different influence factors, such as passing traces, speeds, and noises. At this time, the machine learning classifier can regard these factors as damage indicators and thus make high accuracy values. Additionally, when frequency responses are utilized, the classification capability can decrease because the number of features is much greater than that of samples (runs). Thus, if the raw frequency responses are utilized, the authors would recommend collecting as many “healthy” data as possible to weaken the inverse effects of influence factors. Notwithstanding, even if when the bridge is healthy, high accuracy can be obtained, it can be noticed that if the bridge is damaged, the accuracy will become higher. A clear gap between the accuracy values is noticed. Thus, due to this characteristic of the accuracy values, a threshold can be selected to determine the bridge’s healthy condition. To select a good threshold $k_{s}$, we expect that when the bridge is healthy, 95% accuracy values are less than $k_{s}$, and when the bridge is damaged, 95% accuracy values are greater than $k_{s}$. It can been determined that $k_{s}=0.84$ satisfies both the SSB and CSB models. Nonetheless, the above study employing raw frequency responses are in a space with high dimensions. For example, when frequency responses between 0 and 500 Hz are selected, the classification accuracy becomes steady. However, employing frequency responses in the range of 0–500 Hz results in 6553 features, which poses a challenge for the classifier and necessitates more CPU resources for calculating the classification results. Thus, before using the proposed method in engineering, it is necessary to find ways to reduce the input dimensions of the classifier. In the subsequent sections, various DRTs are studied based on the specified threshold $k_{s}$. An effective DRT needs to keep this characteristic when determining the bridge’s healthy conditions.

### 4.2. Damage Detection Using Different DRTs

In this part, a few of the DRTs are investigated. These techniques are used to reduce the dimensions of raw frequency responses. The frequency range used in this study was 0–5000 Hz. As the vehicle’s speed varies between runs, the length of each signal also differs. To accelerate the processing performance of FFT and to align the frequency responses of the vehicle, time-domain signals were padded with zeros, and their lengths were set to $2^{17}=13,1072$. In the frequency domain, the frequency resolution was 0.0763 Hz, and there were 65,537 frequency points. Different DRTs were used to reduce all 65,537 features to 1–200 dimension spaces. Using the LR classifier, the accuracies of SC1 vs. SC2 and SC1 vs. SC3 (or CC1 vs. CC2 and CC1 vs. CC3) were calculated. As dividing the dataset into different folders is random in the CV process, it is carried out ten times to avoid occasional accuracy.

#### 4.2.1. Damage Detection Using PCA

**Figure 13:**
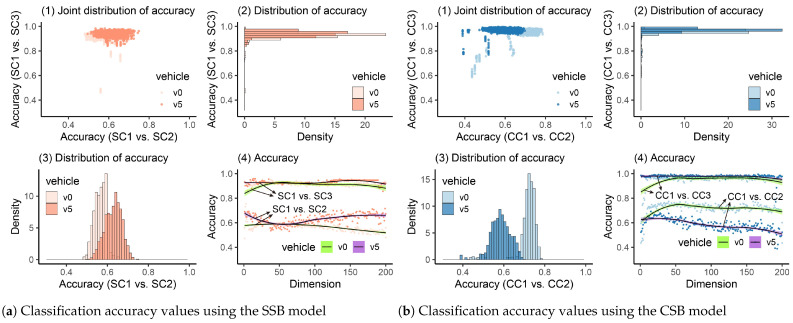
Classification accuracy values using PCA.

After the PCA technique is employed, the classification accuracy values when the vehicle’s frequency responses are reduced to 1–200 latent representations are shown in Figure 13(a4,b4). We can see that when the bridge stays in a healthy state, the accuracy values are lower than 0.8. However, after the bridge is damaged, the accuracy increases a lot. Additionally, we can see that once the heavy vehicle is employed, the accuracy values are higher when the bridge is damaged. Similar results are obtained when the raw frequency responses are utilized, as discussed in Section 4.1. After the CV process was executed ten times, the joint distributions of accuracy values were plotted in Figure 13(a1,b1). From the marginal distribution of accuracy values shown in Figure 13(a2,a3,b2,b3), we can see that for SC1 vs. SC2 or CC1 vs. CC2, the distribution range is large. However, if there is true damage on the bridge (SC1 vs. SC3 or CC1 vs. CC3), the distribution of accuracy values should become thinner. To test the threshold, quantiles were calculated for Table 5. It can be seen that 0.84 can be used to determine the bridge’s healthy condition. However, it was found that PCA is sensitive to the number of training samples. When the samples (runs) are limited (less than 50), even if the bridge is damaged, the classifier may not be able to learn features using principal components in a small space [67]. Thus, when the PCA is employed, the authors would suggest collecting as many samples as possible (e.g., more than 200 runs for each scenario).

#### 4.2.2. Damage Detection Using UMAP

**Figure 14:**
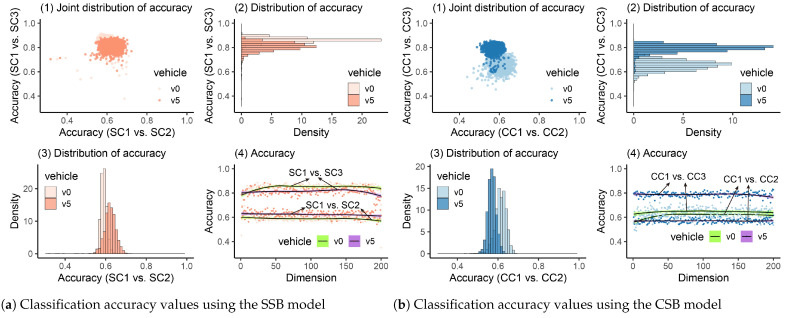
Classification accuracy values using UMAP.

Figure 14 depicts the damage-detection results for the SSB and CSB models when UMAP is utilized. Figure 14(a4,b4) demonstrate that as the dimensions are increased from 1 to 200, the curves of accuracy values for the two beam models are approximate horizontal lines. Additionally, it can be seen that if the bridge is in its healthy state for the SSB model, the accuracy is mainly distributed around 0.6 (see Figure 14(a3)). If there is damage in the bridge, the accuracy stabilizes at near 0.8 (see Figure 14(a2)). Vehicle mass has no apparent effects on damage-detection results. For the CSB model, the occurrence of damage can increase the classification accuracy from 0.5–0.7 to 0.6–0.8. However, even though the accuracy is improved, it cannot meet the threshold $k_{s}=0.84$ determined by raw frequency responses. The quantiles of damage detection accuracy values are shown in Table 6. It can be seen that for neither the SSB nor the CSB model, when the bridge is damaged, the 5% quantiles can reach the threshold. Thus, under this condition, UMAP cannot be used to reduce the input dimensions and further determine the bridge’s health condition using the vehicle’s vibrations.

#### 4.2.3. Damage Detection Using MDS

**Figure 15:**
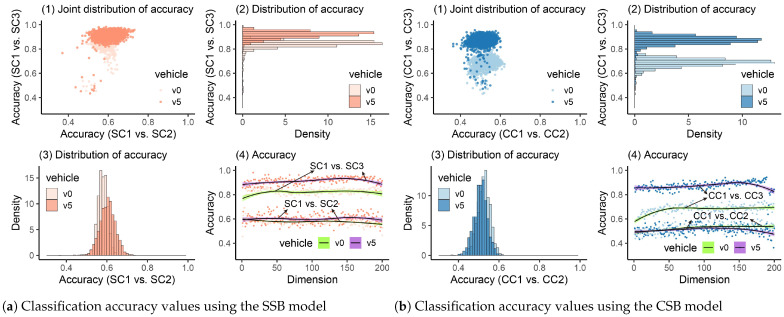
Classification accuracy values using MDS.

The results of damage detection using MDS can be found in Figure 15 for the SSB and CSB models. From Figure 15(a4,b4), we can see that when the used dimensions increase, the accuracy for most scenarios is approximately unchanged, meaning that the MDS does not depend on the number of dimensions used. In Figure 15(a3), when the SSB model is employed, we can see that the vehicle’s weight has little influence when the bridge is healthy. The classification accuracy was distributed around 0.6. If the bridge was damaged, it can be seen in Figure 15(a2) that nearly all accuracy values were greater than 0.8. For the heavy vehicle, the accuracy could reach 1.0 sometimes. For the CSB model, similar results could be observed when the bridge was healthy. The accuracy was mainly distributed between 0.4 and 0.6, and the vehicle’s weight influences accuracy values little. Notwithstanding, when damage occurred in the CSB model, different vehicles could have a great impact on the damage-detection results. We can see that when the vehicle was light (v0), the accuracy decreased to be smaller than 0.8, whereas in the scenario when the heavy vehicle (v5) was utilized, the accuracy could stay relatively high (0.8–1.0). To clearly explore the results when MDS was utilized, we listed all quantiles of accuracy values in Table 7. It can be seen that only when the heavy vehicle and SSB model were utilized, could the accuracy results meet the requirement mentioned in Section 4.1. Therefore, using MDS to determine the bridge’s condition depends on different scenarios when different vehicles or types of bridges are employed. Its universality cannot be ensured, and thus is not suitable for practical engineering when more factors are considered.

#### 4.2.4. Damage Detection Using SAE

**Figure 16:**
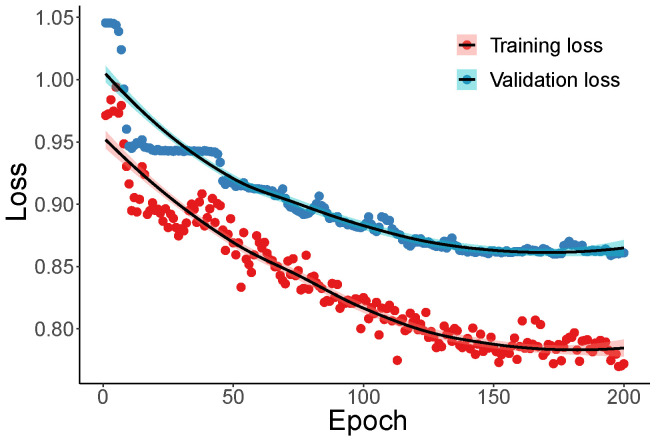
Loss of SAE.

**Figure 17:**
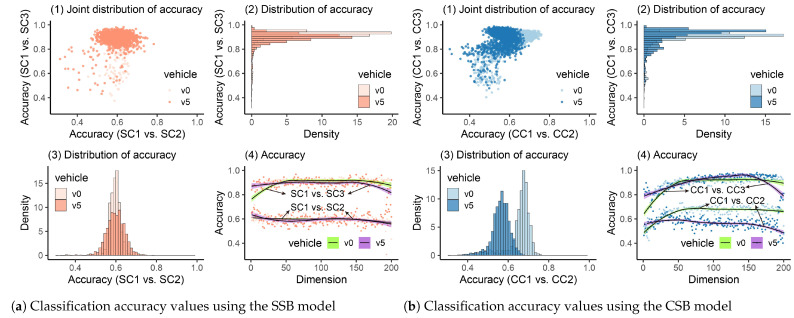
Classification accuracy values using SAE.

SAE is an unsupervised deep learning method, typically used to rebuild the input and keep the most crucial input information in the latent state (bottleneck). Commonly, the latent representations of the input in the bottleneck in a low-dimension space will be utilized to achieve dimension reduction. Before the SAE model is employed, its hyperparameters must be determined. In this paper, the used hyperparameters are listed in Table 8. Additionally, because the initial weights in the SAE were randomly selected, the classification process was executed ten times. Therefore, there were 10 (times) × 4 (scenarios) × 200 (dimensions) × 2 (healthy or damaged) = 16,000 training processes. For each training process, 70% of data were used for training, and 30% of them were utilized for validation. Figure 16 plots the training and validation loss when there were 200 neurons in the bottleneck. The damage-detection results when the SAE was utilized are shown in Figure 17.

In Figure 17(a4,b4), we can see that when the bridge was healthy, the accuracy fluctuated around 0.6 and 0.7. If the bridge was damaged, the accuracy could increase at first when more dimensions were utilized. However, the accuracy became lower when the utilized dimensions improved to more than 150. Regarding the distribution of the accuracy, for the SSB model, the vehicle’s weights had no evident influence on accuracy. If the bridge was damaged, most accuracy values were greater than 0.8; otherwise, if the bridge was healthy, those values were mainly distributed around 0.6. However, for the CSB model, we can see that when the bridge is healthy, different vehicle weights will induce different distributions of accuracy values, as shown in Figure 17(b3). The quantiles of accuracy values are listed in Table 9. We can see that even though for all scenarios, when the bridge was healthy, 95% accuracy values were smaller than 0.84. However, when the bridge was damaged, only the normal vehicle (v0) passing on the SSB model could suit the threshold $k_{s}=0.84$. Thus, the SAE model is not suitable for the problem discussed in this paper. In addition, the trained model using vehicle vibrations when the bridge is healthy cannot be used for the “damage” cases because they may have different patterns. After new vibration data are obtained, modifying hyperparameters to train a new model can require much time, and data processing efficiency cannot be ensured.

#### 4.2.5. Damage Detection Using MFCCs

**Figure 18:**
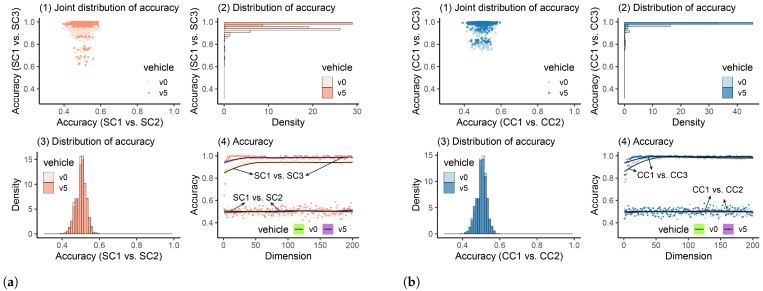
Classification accuracy values using MFCCs. (**a**) Classification accuracy values using the SSB model. (**b**) Classification accuracy values using the CSB model.

MFCCs have been verified to be effective in SHM [68,69,70] but are rarely employed in the indirect method for bridge-health monitoring. The damage-detection results using MFCCs are plotted in Figure 18. In Figure 18(a4,b4), we can observe that when the bridge is healthy, the accuracy values are around 0.5, and no increase or decrease trend can be seen. After the bridge is damaged, for the SSB model, different vehicle weights can cause divergence in accuracy values, but both can reach high accuracy (>0.84). For the CSB model, similarly, when the vehicle’s weight was relatively low, the accuracy was slightly lower than in the scenario when the heavy vehicle was utilized. Furthermore, we can see from the accuracy’s distribution (Figure 18(a3,b3)) that when the bridge was healthy, no matter what the vehicle’s weight was, the accuracy values were intensively distributed between 0.4 and 0.6, which can be clearly regarded as showing that the bridge was in a healthy state. By calculating all scenarios’ quantiles, we can get Table 10.

In Table 10, we can see that when the bridge was healthy, for all four scenarios, 95% accuracy values were lower than 0.54, which is acceptable when the threshold $k_{s}=0.84$ is utilized. After the damage occurred in the bridge, the accuracy became higher in all scenarios, and 95% values can be greater than the threshold. Furthermore, it can also be observed that when the heavy vehicle was used, the accuracy became higher (from 0.89 to 0.98 for the SSB model and from 0.91 to 0.98 for the CSB model). The accuracy difference when the bridge was healthy and damaged was more identifiable. Thus, similarly to the analysis where the raw frequency responses are utilized, a heavier vehicle (around 5% of the bridge’s mass) is recommended when the proposed method is utilized in practical engineering.

The above analysis utilized all “healthy” and “damage” runs to perform damage detection. However, in practical engineering, damage can occur in a specific position with a deterministic degree at a time $T_{i}$. If the cases in Table 2 are analyzed separately, the damage-detection results using MFCCs can be found in Table 11 and Table 12.

It can be seen that when the threshold of 0.84 was utilized, different damage cases could still be identified. Furthermore, we can notice that the lightest damage appeared in case s03 or case s53 when a 2 kg mass was added to the SSB model’s middle point. After employing the accelerations collected by the accelerometer attached to the bridge (direct method) in Figure 6, we saw that the bridge’s fundamental frequency became 35.02 Hz in case s03 and case s53. Compared to the intact bridge, its fundamental frequency decreased by 0.74%. Such a minor change in the bridge’s natural frequency can still be identifiable using the vehicle’s frequency responses, which verifies the high sensitivity of the proposed method to damage.

## 5. Conclusions and Future Work

A promising machine learning-based method called A${}^{2}$M for detecting the bridge’s damage using accelerations of moving vehicles is proposed in this paper. It is independent of adjusting the vehicle parameters and can suit different types of beam bridges. Even though it is supervised, when applied in practical engineering, the labels of damaged cases are unnecessary. For the machine learning model, the vehicle’s full-band frequency responses are typically utilized as the input for training at first. Then, to improve the efficiency and damage detection precision, several dimension reduction techniques can be explored to space the vehicle’s frequency responses into low-dimension spaces for classification. The main conclusions are drawn below:

1. Apart from the vehicle’s frequency responses in a low range (0–50Hz), its full-band frequency responses contain abundant information about the bridge’s health conditions and thus are recommended to be considered in bridge-health-condition identification.
2. When the A${}^{2}$M was employed for bridge-health monitoring, in this study, the threshold was determined as 0.84 to detect the bridge’s damage, which can be used as a reference for similar engineering applications.
3. Among all DRTs, utilizing the threshold determined by raw frequency responses, we found that PCA and MFCCs can work well for the proposed dimension reduction problem. UMAP, MDS, and SAE depend on different scenarios or hyperparameters and may fail sometimes.
4. MFCCs are more sensitive to damage than PCA. This caused the accuracy values to be centered around 0.5 when the bridge was healthy, but the accuracy values became high after the bridge was damaged. The results are beneficial for detecting the bridge’s damage using the A${}^{2}$M.
5. Based on the damage-detection results using the vehicle’s raw frequency responses and MFCCs, a heavy vehicle (>5% of the bridge’s mass) is recommended but not mandatory.

Even though promising results were obtained with the laboratory environments, some special damage types, such as defects of supports and extremely slight damage, were not considered. In addition, the proposed method required the usage of the same vehicle, and possible solutions and the influences of different vehicular characteristics on damage detection deserve further exploration. Improvement of the proposed method can include more factors, e.g., using different vehicles and bridges, considering effects of wind and temperature, and multiple moving vehicles. Scale effects of the laboratory experiments and damage sensitivity will also be investigated before field tests.

## Figures and Tables

**Table 2:** Cases in the experiment.

VBI Model	Scenario 1: SSB+v0	Runs	Scenario 2: SSB+v5	Runs
Runs on healthy bridge	Case s00${}^{*1}$ (healthy)	568	Case s50 (healthy)	562
Runs on damaged bridge	Case s01 (0.3 L${}^{*3}$: 15 kg, 0.6 L: 4 kg)	66	Case s51 (0.3 L: 15 kg, 0.6 L: 4 kg)	66
	Case s02 (0.4 L: 20 kg)	79	Case s52 (0.4 L: 20 kg)	66
	Case s03 (0.5 L: 2 kg)	66	Case s53 (0.5 L: 2 kg)	67
VBI model	Scenario 3: CSB+v0	Runs	Scenario 4: CSB+v5	Runs
Runs on healthy bridge	Case c00 (healthy)	565	Case c50 (healthy)	506
Runs on damaged bridge	Case c01 (0.3 LL${}^{*4}$: 10 kg, 0.3 RL: 20 kg)	56	Case c51${}^{*2}$ (0.3 LL: 10 kg, 0.3 RL: 20 kg)	51
	Case c02 (0.3 RL${}^{*4}$: 30 kg)	58	Case c52 (0.3 RL: 30 kg)	48
	Case c03 (0.5 LL: 5 kg)	56	Case c53 (0.5 LL: 5 kg)	51
	Case c04 (0.5 LL: 10 kg, 0.5 RL: 20 kg)	58	Case c54 (0.5 LL: 10 kg, 0.5 RL: 20 kg)	50

*1 Case s00: s → SSB, 0 → v0, 0 → healthy case; *2 Case c51: c → CSB, 5 → v5, 1 → damage case 1; *3 L: the span of SSB; 0.3L: the mass is attached to the position of 1/3 span; *4 LL: CSB’s left span; RL: CSB’s right span; 0.3LL: the mass is attached to the position of 1/3 CSB’s left span.

**Table 3:** Influence of added mass on the fundamental frequency.

Cases	$f_{\mathrm{SSB},1}$ (Frequency Decrease Ratio)	Cases	$f_{\mathrm{CSB},1}$ (Frequency Decrease Ratio)
Case s00 (Case s50)	35.28 Hz	Case c00 (Case c50)	30.75 Hz
Case s01 (Case s51)	34.56 Hz (2.04%)	Case c01 (Case c51)	27.01 Hz (12.16%)
Case s02 (Case s52)	34.26 Hz (2.89%)	Case c02 (Case c52)	26.55 Hz (13.66%)
Case s03 (Case s53)	35.02 Hz (0.74%)	Case c03 (Case c53)	28.84 Hz (6.21%)
–	–	Case c04 (Case c54)	25.94 Hz (15.64%)

**Table 4:** Determination of threshold of accuracy values using raw frequency responses.

VBI model	Scenario 1: SSB+v0				Scenario 2: SSB+v5			
	SC1 vs. SC2		SC1 vs. SC3		SC1 vs. SC2		SC1 vs. SC3	
Quantile	5%	95%	5%	95%	5%	95%	5%	95%
Accuracy	0.55	**0.64**	**0.86**	0.94	0.65	**0.73**	**0.84**	0.89
VBI model	Scenario 3: CSB+v0				Scenario 4: CSB+v5			
	CC1 vs. CC2		CC1 vs. CC3		CC1 vs. CC2		CC1 vs. CC3	
Quantile	5%	95%	5%	95%	5%	95%	5%	95%
Accuracy	0.74	**0.83**	**0.92**	0.96	0.74	**0.83**	**0.91**	0.98

**Table 5:** Quantiles of accuracy values using PCA.

VBI model	Scenario 1: SSB+v0				Scenario 2: SSB+v5			
	SC1 vs. SC2		SC1 vs. SC3		SC1 vs. SC2		SC1 vs. SC3	
Quantile	5%	95%	5%	95%	5%	95%	5%	95%
Accuracy	0.52	**0.61**	**0.89**	0.95	0.56	**0.69**	**0.91**	0.96
VBI model	Scenario 3: CSB+v0				Scenario 4: CSB+v5			
	CC1 vs. CC2		CC1 vs. CC3		CC1 vs. CC2		CC1 vs. CC3	
Quantile	5%	95%	5%	95%	5%	95%	5%	95%
Accuracy	0.67	**0.76**	**0.93**	0.97	0.51	**0.66**	**0.95**	0.99

**Table 6:** Quantiles of accuracy values using UMAP.

VBI model	Scenario 1: SSB+v0				Scenario 2: SSB+v5			
	SC1 vs. SC2		SC1 vs. SC3		SC1 vs. SC2		SC1 vs. SC3	
Quantile	5%	95%	5%	95%	5%	95%	5%	95%
Accuracy	0.57	**0.62**	**0.82**	0.89	0.58	**0.67**	**0.75**	0.86
VBI model	Scenario 3: CSB+v0				Scenario 4: CSB+v5			
	CC1 vs. CC2		CC1 vs. CC3		CC1 vs. CC2		CC1 vs. CC3	
Quantile	5%	95%	5%	95%	5%	95%	5%	95%
Accuracy	0.56	**0.65**	**0.59**	0.72	0.53	**0.60**	**0.74**	0.84

**Table 7:** Quantiles of accuracy values using MDS.

VBI model	Scenario 1: SSB+v0				Scenario 2: SSB+v5			
	SC1 vs. SC2		SC1 vs. SC3		SC1 vs. SC2		SC1 vs. SC3	
Quantile	5%	95%	5%	95%	5%	95%	5%	95%
Accuracy	0.54	**0.62**	**0.80**	0.88	0.54	**0.67**	**0.86**	0.96
VBI model	Scenario 3: CSB+v0				Scenario 4: CSB+v5			
	CC1 vs. CC2		CC1 vs. CC3		CC1 vs. CC2		CC1 vs. CC3	
Quantile	5%	95%	5%	95%	5%	95%	5%	95%
Accuracy	0.47	**0.58**	**0.63**	0.73	0.45	**0.56**	**0.82**	0.92

**Table 8:** Hyperparameter of the SAE.

Hyperparameter	Values
Neurons in layers	(65,537)→(4096)→(512)→(1-200)→(512)→(4096)→(65,537)
Activation	LeakyReLU
Optimizer	Adam
Learning rate	0.0001
Batch size	128
Regularization: $l_{2}$ penalty	0.001
Epochs	200

**Table 9:** Quantiles of accuracy values using SAE.

VBI model	Scenario 1: SSB+v0				Scenario 2: SSB+v5			
	SC1 vs. SC2		SC1 vs. SC3		SC1 vs. SC2		SC1 vs. SC3	
Quantile	5%	95%	5%	95%	5%	95%	5%	95%
Accuracy	0.56	**0.63**	**0.85**	0.95	0.53	**0.67**	**0.82**	0.94
VBI model	Scenario 3: CSB+v0				Scenario 4: CSB+v5			
	CC1 vs. CC2		CC1 vs. CC3		CC1 vs. CC2		CC1 vs. CC3	
Quantile	5%	95%	5%	95%	5%	95%	5%	95%
Accuracy	0.51	**0.72**	**0.71**	0.94	0.48	**0.63**	**0.77**	0.97

**Table 10:** Quantiles of accuracy values using MFCCs.

VBI model	Scenario 1: SSB+v0				Scenario 2: SSB+v5			
	SC1 vs. SC2		SC1 vs. SC3		SC1 vs. SC2		SC1 vs. SC3	
Quantile	5%	95%	5%	95%	5%	95%	5%	95%
Accuracy	0.46	**0.54**	**0.89**	0.95	0.45	**0.54**	**0.98**	1.00
VBI model	Scenario 3: CSB+v0				Scenario 4: CSB+v5			
	CC1 vs. CC2		CC1 vs. CC3		CC1 vs. CC2		CC1 vs. CC3	
Quantile	5%	95%	5%	95%	5%	95%	5%	95%
Accuracy	0.45	**0.54**	**0.91**	0.99	0.46	**0.54**	**0.98**	1.00

**Table 11:** Quantiles of accuracy values using MFCCs for SSB.

Scenarios	Scenario 1: SSB+v0		Scenario 2: SSB+v5	
VBI model	Case s00 vs. Case s00		Case s50 vs. Case s50	
Quantile	5%	95%	5%	95%
Accuracy	0.46	**0.54**	0.46	**0.54**
VBI model	Case s00 vs. Case s01		Case s50 vs. Case s51	
Quantile	5%	95%	5%	95%
Accuracy	**0.92**	1.00	**0.98**	1.00
VBI model	Case s00 vs. Case s02		Case s50 vs. Case s52	
Quantile	5%	95%	5%	95%
Accuracy	**0.89**	0.98	**0.89**	1.00
VBI model	Case s00 vs. Case s03		Case s50 vs. Case s53	
Quantile	5%	95%	5%	95%
Accuracy	**0.92**	0.98	**0.96**	1.00

**Table 12:** Quantiles of accuracy values using MFCCs for CSB.

Scenarios	Scenario 3: CSB+v0		Scenario 4: CSB+v5	
VBI model	Case c00 vs. Case c00		Case c50 vs. Case c50	
Quantile	5%	95%	5%	95%
Accuracy	0.46	**0.54**	0.45	**0.54**
VBI model	Case c00 vs. Case c01		Case c50 vs. Case c51	
Quantile	5%	95%	5%	95%
Accuracy	**0.96**	1.00	**0.96**	1.00
VBI model	Case c00 vs. Case c02		Case c50 vs. Case c52	
Quantile	5%	95%	5%	95%
Accuracy	**0.93**	0.99	**0.97**	1.00
VBI model	Case c00 vs. Case c03		Case c50 vs. Case c53	
Quantile	5%	95%	5%	95%
Accuracy	**0.97**	1.00	**0.95**	1.00
VBI model	Case c00 vs. Case c04		Case c50 vs. Case c54	
Quantile	5%	95%	5%	95%
Accuracy	**0.91**	1.00	**0.97**	1.00

## Data Availability

The data used to support the findings of this study are available from the corresponding author upon request.
